# Nanoparticle or conventional adjuvants: which one improves immune response against Brucellosis?

**DOI:** 10.22038/ijbms.2019.31748.7642

**Published:** 2019-04

**Authors:** Soheil Yousefi, Tooba Abbassi-Daloii, Mojtaba Tahmoorespur, Mohammad Hadi Sekhavati

**Affiliations:** 1 Department of Animal Science, Ferdowsi University of Mashhad, Mashhad, Iran

**Keywords:** Aluminum hydroxide, Brucellosis, Chitosan, Freund, OMP25

## Abstract

**Objective(s):**

Brucellosis is a common infectious disease among animals and humans. While subunit vaccines could be used as an efficient strategy against pathogens, they usually seem to be less immunogenic than live or killed vaccines. However, the use of a suitable adjuvant accompanied by subunit vaccines can be a good alternative to enhance the immune response.

**Materials and Methods:**

To find a proper adjuvant against Brucellosis, the immune response of induced mice by Aluminum Hydroxide (AH), Incomplete Freund (IFA), and Chitosan Nanoparticle (CS) adjuvants in individuals and in combination with CS were assessed.

**Results:**

Immunization with CS stimulated higher interferon gamma (IFN-γ) immunity, while there were no significant differences between rOMP25 (IFA), rOMP25 (AH), rOMP25 (AH-CS) and rOMP25 (IFA-CS) recombinant proteins. Tumor necrosis factor alpha (TNF-α) analysis revealed there were no significant differencesbetween immunized groups and the positive control group, except for the treatment formulated in single IFA. Furthermore, unlike IFN-γ, there was a reverse interleukin-4 (IL-4) immune response trend for treatments, as rOMP25 (CS) displayed the lowest response. rOMP25 (CS) induced higher titer of total antibody than the other ones. Although the recombinant proteins emulsified in different adjuvants induced similar titer of IgG1 antibody, the ones that were formulated in CS, IFA and IFA-CS showed a higher titer of IgG2a. The cell proliferation assay demonstrating the antigen-specific cell proliferative response could be promoted after immunization with CS.

**Conclusion:**

CS whether single or in combination with IF adjuvants has potential to improve Th1-Th2 responses.

## Introduction

Infectious diseases after cardiovascular ones are the second cause of worldwide deaths (1, 2). Brucellosis, as one of the seven global neglected zoonotic diseases caused by a genus of Gram-negative *Brucella* bacterium, is a common infectious disease between animals and humans (2, 3). Vaccination is one of the most effective public health interventions ever performed and has vast impact on preventing infectious diseases (4, 5). Brucellosis causes many economic and health issues that are not preventable using inoculation (6). Therefore, designing an efficient vaccine candidate that can stimulate a protective immune response against this infection seems to be critical (4). Consequently, the development of efficient vaccines will be through using well matched antigens and adjuvants (5). In the 1920s, the concept of adjuvant, which means to aid, was primarily used by Ramon (3). Adjuvants are substances added to vaccines to increase antigen immunogenicity by contributing to the initiation of the innate immune response (4). The adjuvant enhances the efficacy of the vaccine through increasing the immunogenicity of recombinant proteins, decreasing the amount of antigens needed to form protective immunity, raising functional antibody titer, generating faster and long-lasting immune responses, inducing robust cell-mediated immunity, providing broad protection (cross-reactivity), and as an antigen delivery system improves the antigen uptake (1, 7-9). Adjuvants can be classified based on their physicochemical features, sources, and mechanisms of action (10). Aluminum Hydroxide (AH) and Freund Adjuvants (FA) are two kinds of mineral salt adjuvants and Chitosan (CS) is a sort of mucosal adjuvant that are used in the current study. Glenny *et al*. (11) demonstrated the adjuvant activity of aluminum compounds that were based on absorbed diphtheria toxoid. After around a decade, aluminum-based compounds remained the main used adjuvants (12). The AH mechanism includes the formation of a depot at the vaccination site, enhancing antigen availability and activation of antigen presenting cells (13). However, it is a poor inducer of cell-mediated immune response, which is essential for protection against many pathogens such as Brucellosis and it is important to increase allergenicity and neurotoxicity as well (14-18). So after Alum, Complete Freund’s Adjuvant (CFA), which consists of heat-killed *Mycobacterium tuberculosis* was developed (19, 20). CFA is a feasible adjuvant, although it is too toxic and causes severe local reactions in humans. Therefore, Incomplete Freund (IFA), which lacks killed *Mycobacterium *found in CFA, was introduced (20, 21). It stimulates predominantly Th2 response through the formation of a depot at the injection site, enabling the gradual antigen release and stimulating antibody to produce plasma cells (22). IFA is routinely used for boosting immunizations subsequent to CFA (23). CS is produced using the deacetylation of chitin, the major element of the shells of crustaceans (24, 25). CS as a nontoxic immunological adjuvant can induce both humoral and cell-mediated immunity by stimulating macrophages, natural killer cells, dendritic cells, and B and T lymphocytes (26-33). Also, it can be used as a vaccine delivery carrier (34).

Herein we assumed that CS can have a better potential to promote an immune response against *Brucella* by stimulating the innate immune system. In this regard, the immune responses of three different adjuvants were assessed when they were injected either individually or in combination with CS. OMP25 was used as a model antigen because it is the major antigen involved in the survival of *Brucella* and it is also highly conserved among different *Brucella* species (35).

## Materials and Methods

***Bacterial strains and recombinant protein productions***

*B. melitensis* strain Rev 1 was obtained from the *Brucella* culture collection of Razi Vaccine and Serum Research Institute (Mashhad, Iran), and cultured as described (36). The OMP25 recombinant protein was produced as previously described (35). 

**Figure 1:**
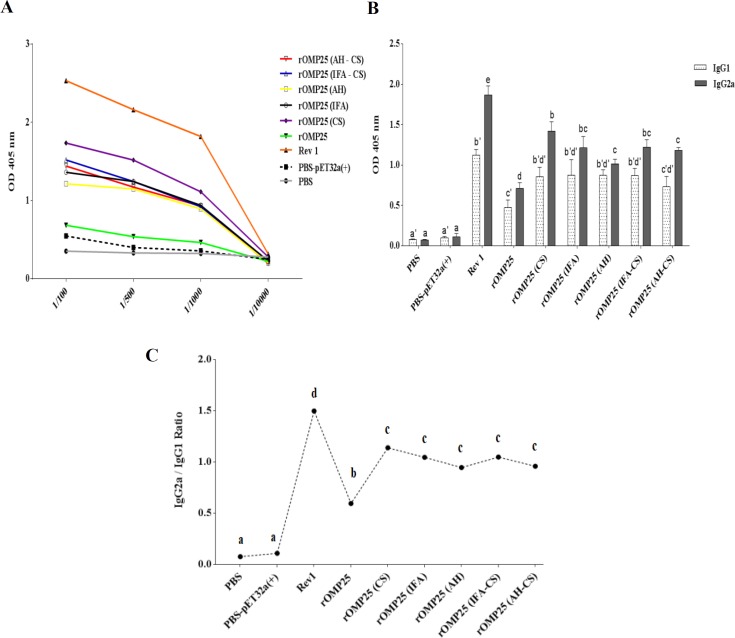
Kinetics production of antibody

A) Total antibody titer for each treatment in different adjuvants measured at OD_405_ nm with an ELISA reader. B) IgG1 and IgG2a responses in immunized mice. C) The ratio of IgG2a to IgG1 in immunized mice. Levels of each antibody were measured at OD405 nm with an ELISA reader. Each value represents the mean of triplicates±SE of antibody responses from five samples. Different letters indicate statistically significant differences between experimental groups (*P<*0.05), where there are same letters it means that those groups induced the same level of antibody and there were no statistically significant differences between them and vice versa. rOMP25 refers to the baseline group, PBS and PBS-pET-32a ^(+)^ refer to negative control groups. Rev1 refers to live attenuated vaccine *B. melitensis *Rev1 as a positive control group

**Figure 2:**
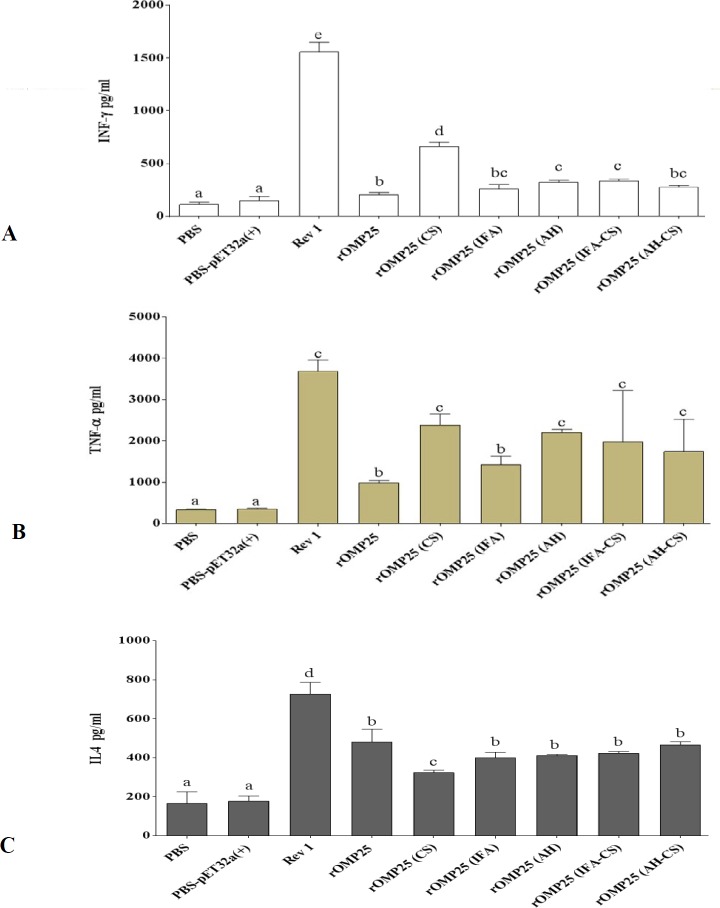
Determination of cytokine responses in spleen cells from immunized mice

A) IFN-γ response from immunized mice with different adjuvants. B) TNF-α response from immunized mice with different adjuvants. C) IL-4 response from immunized mice with different adjuvants. Levels of each cytokine were quantified (pg/ml) by ELISA at OD_405_ nm. Each value represents the mean of triplicates±SE of antibody responses from five samples. Different letters indicate statistically significant difference between experimental groups (*P<*0.05), where there are same letters it means that those groups induced the same level of antibody and there were no statistically significant differences between them and vice versa. rOMP25 refers to the baseline group, PBS and PBS-pET-32a^(+)^ refer to negative control groups. Rev1 refers to live attenuated vaccine *B. melitensis* Rev1 as a positive control group

**Figure 3:**
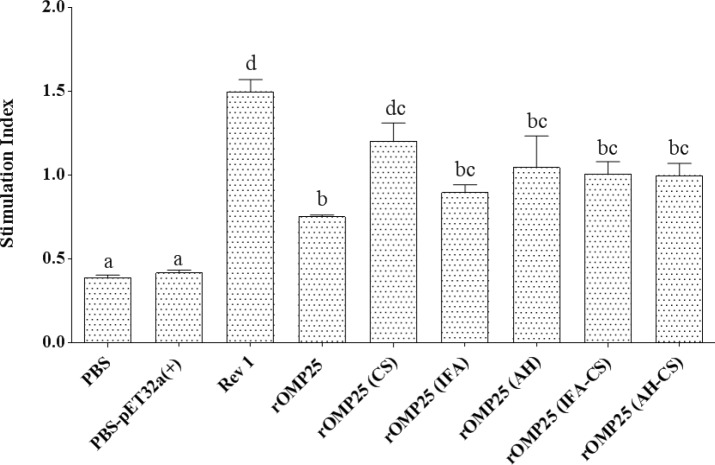
Lymphocyte proliferation responses of the experimental groups after *in vitro* antigen recall. The stimulation indexes of the experimental groups are shown as mean of triplicates ± SE from five samples. Different letters indicate statistically significant differences between experimental groups (*P<*0.05), where there are same letters it means that those groups induced the same level of antibody and there were no statistically significant differences between them and vice versa. rOMP25 refers to the baseline group, PBS and PBS-pET-32a^(+)^ refer to negative control groups. Rev1 refers to live attenuated vaccine *B. melitensis* Rev1 as a positive control group

***Experimental groups and immunization***

Six-week-old female BALB/c mice (obtained from Razi Vaccine and Serum Research Institute, Iran and kept according to institutional policies for animal health and welfare) were randomly classified into nine experimental groups containing rOMP25, rOMP25 (CS), rOMP25 (IFA), rOMP25 (AH), rOMP25 (AH-CS), rOMP25 (IFA-CS), *B. melitensis* Rev 1, PBS - pET-32a^(+) ^and PBS (5 mice per group). As immunization was performed intraperitoneally (IP) with OMP25 recombinant protein (20 µg per injection) emulsified in three different adjuvants including individual injections of AH, IFA and CS with concentration of 0.5 mg/ml for each one (Sigma, USA) or combination of AH and IFA with CS. Additionally, mice were administered rOMP25 without adjuvant, which was used to determine baseline immune responses elicited by antigen alone and to correct the probable impact of this antigen on immune responses to get unbiased results. Therefore, different levels of stimulated immune responses are expected due to different adjuvants. Moreover, there were two different negative control groups including phosphate buffered saline (PBS) and PBS containing self-expressed pET-32a^(+)^ vector. A dose of live attenuated vaccine *B. melitensis* Rev1 (10^6^ CFU/mice) was injected as positive control group. PBS was added to each vaccine mixture to a final volume of 300 μl per injection. All experimental groups were immunized three times (days 0, 15, and 30).

***Antibody and isotype responses***

Mice were bled for serum collection two weeks after the last injection. Serum was separated from blood by centrifugation at 3000 g for 20 min and the supernatant was used to determine humoral response using an indirect enzyme-linked immunosorbent assay (ELISA). The purified rOMP25 (1 µg/ml) protein was coated in 96-well plates (Nunc, Naperville, IL) and incubated for 24 hr at 37 ^°^C. Wells were washed three times with PBS containing 0.05% Tween 20 (TPBS) and blocked for 1 hr at 37 ^°^C with 5% skimmed milk in PBS. Plates were afterward incubated with serial dilutions of mouse sera (1/100 to 1/10000) for 2 hr at room temperature and washed three times as above. Then wells were incubated with 100 µl of 1/10000 dilution of anti-mouse IgG–Horseradish Peroxidase (HRP) conjugate antibody (Sigma, USA) for 2 hr at 37 ^°^C. Plates were washed five times and incubated for 15 min with 100 µl of 3, 3’, 5, 5’-tetramethyl-benzidine (TMB) substrate in the dark and the reaction was stopped using 2N H_2_SO_4_. Finally, color intensity was measured at OD_405 _nm with an ELISA plate reader. To determine the Th1/Th2 immune response bias, IgG isotyping was performed under the same condition using 100 µl of 1/4000 dilution of goat anti-mouse IgG1-HRP and IgG2a-HRP conjugated antibodies (Sigma, USA). The results for IgG and IgG isotypes were represented as the mean of triplicates ± SE of the OD_405_ nm from five samples.

***Cytokines determination***

To evaluate the cellular immune response induced by different immunization strategies, mice were sacrificed two weeks after the final injection and their spleens were homogenized in 10 ml PBS containing 5 mM ethylenediamine-tetraacetic acid (PBS-EDTA) on ice. The cells were washed twice with PBS-EDTA and mononuclear cells were isolated. They were then cultured in RPMI 1640 (supplemented with 4 mM l-glutamine, 100 U/ml penicillin, 100 µg/ml streptomycin, and 10% heat-inactivated FBS) at 37 ^°^C in 5% CO_2 _(37). Splenocytes were then counted and a total number of 4×10^6^ cells were seeded in a 24-well plate and stimulated *in vitro* with 10 μg/ml of recombinant protein emulsified in different adjuvants for 48 hr at 37 °C in 5% CO_2_. In order to measure the different cytokine levels, cell culture supernatants were collected and centrifuged at 300 g for 10 min and stored at -80 ^°^C. Interferon-gamma (IFN-γ), tumor necrosis factor alpha (TNF-α), and interleukin-4 (IL-4) levels were measured by sandwich ELISA according to the manufacturer’s instructions (Mabtech, Nacka, Sweden).

***Lymphocyte proliferation assay***

As described previously in the cytokines determination section, spleens were dissected from the mice and suspended in cold sterile PBS containing 2% FBS. RBCs were lysed using lysis buffer. The single-cell suspension was adjusted to 3×10^6^ cells/ml and afterward supplemented in RPMI 1640. Then the cell suspension was dispensed into a 96-well plate in triplicate and incubated with 10 µg/ml of the recombinant protein as antigen recall for 48 hr. Before the incubation ended, 20 µl MTT (3-(4,5 dimethylthiazole-2yl)-2,5 diphenyl tetrazolium bromide, 5 mg/ml) was added. After 4 hr, 100 µl of dimethyl sulfoxide (DMSO) was added followed by 1 hr incubation. Absorbance was measured using a spectrophotometric plate reader at 590 nm. The stimulation index (SI) was calculated according to the following equation: average OD of stimulated wells/average OD of un-stimulated wells.

***Statistical analysis***

Data were analyzed using one-way analysis of variance (ANOVA), followed by Tukey`s *post hoc* test analysis for multiple comparisons using GraphPad Prism v6.07 software (GraphPad Software Inc., San Diego, CA, USA). Values were presented as mean±SE in both text and figures. *P-values* less than 0.05 were considered statistically significant.

## Results

***Chitosan nanoparticle adjuvant increased humoral immune response***

To evaluate the humoral immune response elicited by different immunization strategies, immunoglobulin G (IgG) antibodies were measured using specific indirect ELISA. The result of total antibody response showed immunization with different adjuvants raised higher levels of antibody titer compared to the negative control groups (Figure 1A). Mice immunized with rOMP25 (CS) showed higher total antibody titer than other groups, while it was still lower than the positive control group (Figure 1A). Furthermore, other treatments that were formulated in different adjuvants stimulated a roughly similar total antibody level (Figure 1A). Moreover, IgG1 and IgG2a were evaluated as Th2 and Th1 response markers, respectively. The results illustrated that all treatments elicited higher levels of IgG1 as well as IgG2a antibodies compared with negative control groups (*P*<0.05, Figure 1B). There were no statistically significant differences in IgG1 titer between treatments that were formulated in different adjuvants (rOMP25 (CS), rOMP25 (IFA-CS), rOMP25 (IFA), rOMP25 (AH) and rOMP25 (AH-CS)); additionally, the positive control group showed no statistically significant difference with other treatments that were emulsified in different adjuvants (except rOMP25 (AH-CS)) (*P*<0.05, Figure 1B). The titers of IgG2a antibody in all treatments that were formulated in adjuvants were also higher and lower than negative and positive control groups, respectively. rOMP25 (AH) and rOMP25 (CS) vaccines formed the lowest and the highest amounts of IgG2a antibody titer, respectively among emulsified treatments in adjuvants (*P*<0.05, Figure 1B). Moreover, the antibody titer demonstrated skew from IgG1 to IgG2a in immunized mice indicating strong bent of Th1 immune response, however, there were no significant differences between groups immunized with adjuvants, but they showed statistically higher IgG2a/IgG1 ratio than baseline and negative control groups as well (Figure 1C). Also, rOMP25 (CS) showed the biggest shift from IgG1 to IgG2a among immunized groups with different adjuvants (rOMP25 (IFA-CS), rOMP25 (IFA), rOMP25 (AH), and rOMP25 (AH-CS)), and the average IgG2a/IgG1 ratio was estimated ~ 1.2 (Figure 1C). 

***Immunization with chitosan nanoparticle adjuvant efficiently induced cytokine responses***

Results revealed that all immunizations induced higher titers of both Th1 and Th2 cytokines than negative control groups (Figure 2). The vaccine that was only emulsified in the CS adjuvant (rOMP25 (CS), induced significantly higher titer of INF-γ cytokine response than other immunized groups, although it was still lower than the positive control group (*P*<0.05, Figure 2A). There were no further statistically significant differences between rOMP25 (IFA), rOMP25 (AH), rOMP25 (IFA-CS), and rOMP25 (AH-CS) treatments; rOMP25 (IFA) and rOMP25 (AH-CS) showed no statistical difference with the group treated without adjuvant (rOMP25) (*P*<0.05, Figure 2A). TNF-α analysis exhibited no significant differences between immunized treatment using emulsification in adjuvants and the positive control group, although rOMP25 (IFA) showed statistically no difference compared to rOMP25 (Figure 2B). rOMP25 (CS) showed the lowest IL-4 titer among formulated treatments, whilst there were no significant differences between other immunized groups for IL-4 cytokine (Figure 2C). Also, the positive control group induced the highest titer of IL-4 among all immunization schemes (Figure 2C). Generally, treatments formulated in different adjuvants induced higher titers of both Th1 and Th2 cytokine responses compared with negative control groups, as rOMP25 (CS) and rOMP25 (AH), slightly, showed better efficiency than other treatments. 

***Lymphocyte proliferation***

Lymphocyte proliferation index is increased if the absorbance values of the experimental samples are higher than that of the negative control group and vice versa. In this regard, lymphocyte proliferation index was measured after stimulating splenocytes of immunized mice *in vitro*. There was no difference between the positive control group and rOMP25 (CS), whilst the former of which had a significant difference with other groups (*P*<0.05, Figure 3). Finally, treatments containing CS showed better performance than other groups with different adjuvants, whilst it was not significant.

## Discussion

Vaccination is considered the most efficient and cost-effective tool to prevent a variety of infectious pathogens, whilst inducing a strong immune response to provide long-term protection still remains the main issue. To overcome this problem, using an appropriate adjuvant with vaccines would be helpful (1, 2). Several studies showed alum, IFA and CS could activate the innate immune response, whilst in some cases, consumption of both alum and IFA showed strong side effects in humans and animals, likewise, Alum could not efficiently induce an immune response when it was used alone (38-44). Therefore, for the first time in the current study to find the best adjuvant combination against Brucellosis, the impact of different adjuvants on improving the humoral and cellular immune responses was evaluated. Furthermore, since other adjuvants such as alum did not have proper efficiency in oral immunization (38, 39), the IP route was performed to consider all groups under the same conditions.

The comparison results of AH, IFA, and CS showed immunization with CS (rOMP25 (CS)) not only induced better humoral and cellular immunity but also in some cases there were no statistically significant differences between rOMP25 (CS) and positive control groups (Figures 1 and 2). rOMP25 (AH) stimulated cellular immunity more than humoral immunity, while rOMP25 (IFA) showed the opposite performance compared with rOMP25 (AH). Moreover, the rOMP25 (IFA-CS) group showed slightly higher cellular responses (but not significant) than individual injection of IFA, although there was a reverse immunity response level for rOMP25 (AH-CS) treatment compared with the group with rOMP25 (AH) injection. rOMP25 (IFA-CS) and rOMP25 (AH-CS) injections showed no enhancement to humoral responses compared with individual inclusion of IF and AH (Figure 2). The cell proliferative response indicated vaccination with OMP25 elicits a vigorous antigen-specific cell proliferative response that could be promoted after the immunization with CS and this would be vital for controlling Brucellosis. Overall, in the current study immunization with rOMP25(CS) showed better performance in inducing IFN-γ, TNF-α, IgG2a, and lymphocyte proliferation index and reduced IL-4, indicating the tendency of this adjuvant to stimulate Th1 immune response. The specifically related to CS results of the current study can be explained through functional aspects of IFN-γ and TNF-α, which are two important components of Th1 immune response contributing to controlling Brucellosis by stimulating phagocytic activity of macrophages and apoptosis in infected macrophages (45, 46). The development of both CD4+ (secreting IFN-γ upregulating the anti-*Brucella *activity of macrophages) and CD8+ (secreting IFN-γ and lysing *Brucella*-infected cells) T cells are two main protective responses in the host (47). Moreover, IFN-γ plays a key role in the switching of IgG to IgG2a, which is important in protecting against infection (47). Also, secretion of IFN-γ from CD4+ cells is down-regulated by IL-4 causing exacerbation of the *Brucella* infection (48, 49). 

Our results were in agreement with a study (42) that found antigens emulsified in IFA induced higher antibody titers than injection of antigens without adjuvants in a human influenza vaccine. Additionally, our results are consistent with another study (50) that reported CS noticeably enhanced both cellular and humoral immune responses and stimulated a balanced Th1/Th2 response. That study suggested CS would be a safe and efficient adjuvant candidate for a wide spectrum of prophylactic and therapeutic vaccines. Recently, researchers (51, 52) used CS as an adjuvant in their studies and found a remarkable improvement in immune responses against Brucellosis. Some (53, 54) studied the effect of IFA and *N*-Trimethyl chitosan (TMC) on Omp19 immunogenicity against *Brucella abortus* and *Brucella melitensis*. They found Omp19-IFA and TMC/Omp19 could induce Th1 and Th2 immune responses, respectively, while TMC/Omp19 immunization induced a mixed Th1/Th17 immune response. In addition, a study (55) found that *N*-Trimethyl chitosan nanoparticles loaded with influenza subunit antigen-stimulated higher titer of IgG and it could be a feasible new delivery system for influenza antigens. There exist further studies showing the potential of the CS adjuvant, which are also in agreement with the results of the present study. Carroll *et al*. (44) indicated CS elicits dendritic cells and Th1 responses to promote cellular immunity by engaging the DNA sensor cGAS-STING pathway. They also revealed that CS would be a suitable alternative for alum, although the adjuvanticity mechanism is still underlying (56). Moreover, in contrast with Alum, CS lacks inhibition impact on interleukin-12 production. Additionally, antigen-specific Th1, as well as Th17 responses, were promoted by CS and they have key roles in enhancing immunoglobulin G2c antibody responses (57-59). 

## Conclusion

 In as much as there is no efficient subunit vaccine with a suitable immune response against Brucellosis, CS as an adjuvant individually can improve stimulation of Th1 response, which is essential to deal with infectious diseases. However, the protection efficiency of CS should be examined in further experiments involving domestic animals. 
